# WHO recommendations for pregnancy and postpartum care should include hypertensive disorders of pregnancy 

**DOI:** 10.2471/BLT.25.295382

**Published:** 2026-04-16

**Authors:** Andrea Solnes Miltenburg, Albert Kihunrwa, Richard Kiritta, Joyce L Browne, Benedikte V Lindskog, Johanne Sundby, Joseph R Mwanga, Anne Cathrine Staﬀ

**Affiliations:** a Department of Community Medicine and Global Health, Institute of Health and Society, Faculty of Medicine, University of Oslo, Postboks 1130 Blindern, 0318 Oslo, Norway.; b Department of Obstetrics and Gynaecology, Bugando Medical Center, Mwanza, United Republic of Tanzania.; c Department of Obstetrics and Gynaecology, Catholic University of Health and Allied Sciences, Mwanza, United Republic of Tanzania.; d Department of Global Public Health and Bioethics, Julius Center for Health Sciences and Primary Care, University Medical Center Utrecht, Utrecht University, Utrecht, Kingdom of the Netherlands.; e Department of International Studies and Interpreting, Oslo Metropolitan University, Oslo, Norway.; f School of Public Health, Catholic University of Allied Science, Mwanza, United Republic of Tanzania.; g Institute of Clinical Medicine, University of Oslo, Oslo, Norway.

The World Health Organization’s (WHO) recommendations for antenatal and postpartum care are based on a rigorous development process oﬀering evidence of high-quality essential care, and inform relevant health-care policies and clinical protocols globally. The recent *Toolkit for adaptation of the WHO recommendations for a positive pregnancy and postnatal experience* oﬀers useful guidance.^1^ We recognize that this toolkit provides a valuable response to the need to close the persistent gap between knowledge and action for health, also known as the know-do gap, which causes suboptimal implementation of available evidence. Poor implementation of evidence-based guidelines contributes to continuing high levels of maternal and perinatal mortality and morbidity, especially in low-resource settings.^2^ In this article, we question, however, if the prioritization within these guidelines or toolkits themselves, or lack thereof, sometimes creates implementation challenges and can negatively affect maternal and fetal health outcomes. For example, we question why recommendations for detection, prevention and care for women with hypertensive disorders of pregnancy, including pre-eclampsia, are missing in the WHO recommendation for antenatal and postnatal care and related toolkit.

We are concerned that the chosen toolkit approach, with an emphasis on universal applicability of globally developed indicators and evidence, does not adequately consider implementation challenges and contextual needs. This consideration is important, as insuﬃciently contextualized guidelines may be poorly aligned with existing local priorities, which partially explains why available global recommendations and guidelines do not necessarily lead to improvements in clinical practice and outcomes.^3^ Adoption, adaptation and implementation of such potentially unﬁt guidelines might lead to the unintended consequence of suboptimal implementation, poor prioritization and selective uptake. Moreover, unattainable care standards can also demoralize health workers and may inadvertently cause harm.^4^ National policy-makers rely on global recommendations because they often lack resources to develop their own, and local adaptation is further complicated by the absence of formal guideline development procedures.^5^ Procurement or stock-out challenges, and lack of accountability and transparency mechanisms further hinder effective implementation.^6^ In addition to these challenges, many stakeholders also consider WHO recommendations to be unclear, overwhelming, technically challenging and not always reﬂecting the needs of health workers and communities.^7^

## Where is pre-eclampsia?

In this toolkit, we discern that more consistent integration of evidence across relevant guidelines and regular updates can be strengthened. We illustrate our argument by pointing out the missing attention for hypertensive disorders of pregnancy, including pre-eclampsia, in both the 2016 *WHO recommendations on antenatal care for a positive pregnancy experience*^8^ and the 2025 toolkit.^1^ These disorders are estimated to aﬀect up to 10% of pregnancies and are among the leading causes of maternal mortality and adverse perinatal outcomes worldwide.^9^^,^^10^ Therefore, the absence of reference in the 2025 toolkit to updated evidence-based guidance for risk screening, prevention and management for pre-eclampsia is concerning. Moreover, this lack may be reﬂective of a larger guideline gap: the WHO recommendation for pre-eclampsia prevention and treatment is about 15 years old,^10^ despite the recent scientiﬁc advancements in screening, prevention, diagnosis and management of hypertensive disorders of pregnancy. Meanwhile, the WHO recommendations on antiplatelet agents for the prevention of pre-eclampsia was released in 2021.^11^ This recommendation explicitly oﬀers a suggestion to identify risk factors during antenatal care, as well as women eligible for receiving aspirin prophylaxis. The *WHO recommendations on antiplatelet agents for the prevention of pre-eclampsia*^11^ advise adapting or complementing the list of risk factors (Box 1) based on the local epidemiology of pre-eclampsia, yet there is no reference to this need in the recently published toolkit.

Box 1 **Pre-eclampsia risk factors**

**Moderate risk factors**

- Primiparity
- Family history of pre-eclampsia
- Older than 40 years
- Multiple pregnancy

**High risk factors**

- Diabetes
- Chronic or gestational hypertension
- Renal disease
- Autoimmune disease
- Positive uterine artery Doppler
- Previous history of pre-eclampsia
- Previous fetal or neonatal death associated with pre-eclampsia

Source: WHO, 2021.^11^

The observation that this omission is not incidental, but persistent, is strengthened by our conclusion that the 2016 *WHO recommendations on antenatal care for a positive pregnancy experience* do not include a recommendation for pre-eclampsia within the interventions for maternal and fetal assessment (Category B).^8^ The guideline instead generally states that antenatal screening for pre-eclampsia is an essential part of good antenatal care, which is routinely performed by measuring maternal blood pressure and checking for proteinuria at each antenatal care contact and upon detection of pre-eclampsia, in addition to nutritional intervention for calcium supplements. This notion is repeated in the 2025 toolkit but oﬀers little guidance for health workers regarding how to manage women with hypertensive disorders of pregnancy including pre-eclampsia. In addition, earlier versions of WHO maternal and newborn guidelines oﬀer limited information on these disorders for routine care management, prioritizing instead emergency situations. As a potential consequence, excluding these conditions from the main categories of interventions for maternal and fetal assessments might signal to national governments and providers that blood pressure measurement and checking urine for proteinuria are of lesser importance. Several publications in the past decades have indeed shown that blood pressure measurement and urine analysis during antenatal care are insuﬃciently done both in terms of quality and frequency, irrespective of the antenatal care model.^12^ Therefore, we question why is blood pressure measurement recommended during antenatal care, but antihypertensives are not part of essential medicines listed, as is the case with iron tablets? Why is magnesium sulfate listed but antihypertensive medication is not, rather than both recommended given their distinct and complementary indications? What should health workers do in the absence of blood pressure measurement equipment or urine dipsticks? What should health workers do if blood pressure is high? What should women do if they cannot aﬀord antihypertensive medication?

The toolkit would have oﬀered a good opportunity to re-emphasize the importance of antenatal care to detect and timely manage hypertensive disorders of pregnancy including pre-eclampsia, even in the absence of one uniform updated WHO recommendation for detection, prevention and management of these conditions. More speciﬁcally, with reference to the 2016 WHO antenatal care recommendations,^8^ three key messages could have been considered when the toolkit was developed. 

First, maternal and fetal assessment. Country teams should be encouraged to prioritize recommendations according to the burden of maternal and newborn care in the given context. In settings with high maternal and perinatal mortality due to hypertensive disorders of pregnancy including pre-eclampsia, country teams should explicitly prioritize the assessment of risk screening for pre-eclampsia, provision of prophylaxis and regular measurement of blood pressure and urine analysis. 

Second, preventive measures. In addition to calcium supplementation, in contexts where low dietary calcium uptake is prevalent, the toolkit could have referred to the 2021 WHO antiplatelet guideline to recommend screening for risk factors for pre-eclampsia and women’s eligibility for prophylaxis of low-dose aspirin.^11^ In addition to aspirin, all essential antihypertensive medications could have been added to the medicines listed in the toolkit’s supplementary materials.

Third, management. Upon diagnosis of hypertensive disorders of pregnancy including pre-eclampsia, and with consideration for the level of care and available resources and skills, adequate management of high blood pressure is recommended. The toolkit could have referred to the 2011 WHO guideline for prevention and treatment of pre-eclampsia in terms of choices of medication, preferably with information on doses, route of administration, frequency and recommended follow-up.^10^ In addition, the toolkit could have mentioned a birth plan ensuring timely referral and consideration of induction of labour. 

These additions would have given valuable support to countries with high pre-eclampsia-related maternal and perinatal deaths, oﬀering some guidance to health workers on how to ensure that women with such high-risk pregnancies are oﬀered quality care by health workers who are skilled and able to deal with hypertensive disorders of pregnancy including pre-eclampsia.

Considering the complexities of hypertensive disorders of pregnancy diagnosis and the changing deﬁnitions of pre-eclampsia over the years, updating WHO guidelines on these disorders would send a clear signal to policy-makers and health workers on their importance for maternal and fetal assessment. While many maternal conditions mentioned in the 2016 recommendations^8^ are of course important, none of these contribute as much to maternal and perinatal mortality as hypertensive disorders of pregnancy including pre-eclampsia do. For example, human immunodeficiency virus, tuberculosis, tobacco and substance use, for which the main recommendations for interventions were listed in the 2016 recommendations and repeated in the 2025 toolkit.^1^ While we recognize that care during pregnancy and birth should encompass more than only promoting survival, and focus on the well-being of mother and child, not including hypertensive disorders of pregnancy including pre-eclampsia in the toolkit is a missed opportunity that neglects the high burden of these conditions worldwide, particularly in low- and middle-income settings.
